# COVID-19: Lessons from Norway tragedy must be considered in vaccine rollout planning in least developed/developing countries

**DOI:** 10.1515/med-2021-0334

**Published:** 2021-08-11

**Authors:** Razmin Bari, Asim Kumar Bepari, Hasan Mahmud Reza

**Affiliations:** College of Arts and Sciences, University of North Carolina at Chapel Hill, NC, USA; Department of Pharmaceutical Sciences, North South University, Bashundhara, Dhaka 1229, Bangladesh

**Keywords:** COVID-19 vaccine, elderly population, comorbidities

## Abstract

All governments, regulatory authorities, and vaccine-related committees are under tremendous pressure to roll out vaccines to safeguard the people against COVID-19. To be noted that all COVID-19 vaccines have been developed hurriedly, some with new technologies being used on humans for the first time ever. Most clinical trials did not include elderly patients with comorbidities, hence a careful and logical rollout planning, especially for elderly people, is necessary.

Dear Editor,

When many countries are busy with the rollout process of vaccine to tackle the COVID-19, the sudden death of 71 people after being immunized with Pfizer-BioNTech’s COVID-19 vaccine (BNT162b2) in Europe [1,2,3,4] led us to think critically on this issue. In Norway, over 2 million people received the first dose and 1.36 million people received the second dose of COVID-19 vaccines as of June 15, 2021, and the Norwegian Adverse Drug Reaction (ADR) registry recorded 1,472 reports of possible serious adverse events, including 185 fatality cases [3]. Norway dropped the Oxford-AstraZeneca vaccine from the immunization program [5] following a study by Pottegård et al. [6] reporting a small elevated risk of venous thromboembolic events among people who received the first dose of Oxford-AstraZeneca vaccine ChAdOx1-S in Norway and Denmark. Intriguingly, an expert review published on May 19, 2021 indicated a possible link between COVID-19 immunization and at least ten deaths among elderly frail patients living in Norwegian nursing homes [7]. It has been speculated that the common side effects might have been intensified by the comorbidities in these very old people aged more than 75 years; however, the available evidence is insufficient to draw any causal relationship. Investigations are underway in France, where five frail patients died, apparently coincidentally, after vaccination among 139 reported adverse events [8]. Ten similar death cases have also been reported in Germany. Although the clinical trial results demonstrate that the BNT162b2 vaccine is safe and effective [9], it must not be outright rejected that previously unrecognized adverse reactions might occur during mass immunization using a modern mRNA vaccine. Deaths apparently linked to COVID-19 vaccination are very likely to negate confidence of mass people on vaccines and flag growing concerns that a careful strategy has to be devised for safe use of these vaccines.

Unlike developed countries, there is lack of demographic database of the population that reflects detailed information about people, health, and disease conditions in many Asian and African nations [10,11]. The World Health Organization (WHO) noted that data are scarce on the safety and efficacy of COVID-19 vaccines in immunocompromised patients [12]. Therefore, careful selection of elderly people with comorbidities, stages of the diseases, ongoing treatments, or immune system dysfunctions will be a big challenge in many developing countries. Furthermore, people of least developed/developing countries have inadequate health literacy. In most developing countries, a vast number of non-medical health workers are involved in drug dispensing to the patients. Because of insufficient knowledge, training, and infrastructure, ADR reporting often remains flouted. On the other hand, patients are also ignorant. The regulatory bodies do not have enough capacity to monitor and analyze the ADR data necessary to identify, assess, and publicize ADRs. Smartphone-based apps may help collect ADR responses, but all people do not have the required gadgets, knowledge, and internet coverage. Thus, the real-time data collection and necessary ADR reporting remain obscured. Again, informed consent is not routinely taken from vaccine recipients.

In the above background, many unforeseen casualties may jeopardize the costly vaccination efforts in most of the least developed/developing countries. Also, the robust monitoring and surveillance after immunization, and quick and authentic data sharing across the world necessary for issuing the full use authorization of any specific vaccine are likely to be hampered. Hence, a very careful examination of elderly patients with comorbidities before bringing them into vaccination is warranted. Moreover, the ADR reporting should be practiced to find any safe and effective COVID-19 vaccine. An evidence-based, scientific, and unbiased decision has no alternative.
